# The mitochondrial genome of the endemic Brazilian paradoxical frog *Pseudis tocantins* (Hylidae)

**DOI:** 10.1080/23802359.2018.1508385

**Published:** 2018-10-26

**Authors:** Kaleb Pretto Gatto, Jeramiah J. Smith, Luciana Bolsoni Lourenço

**Affiliations:** a Laboratory of Chromosome Studies, Department of Structural and Functional Biology, Institute of Biology, University of Campinas, Campinas, Brazil;; b Department of Biology, College of Arts and Sciences, University of Kentucky, Lexington, USA

**Keywords:** Mitochondrion, mtDNA, Anura, next-generation sequencing, paradoxical frog

## Abstract

In this work, we present for the first time the mitochondrial genome of a paradoxical frog (*Pseudis tocantins*). This genome is 15.56 kb, excluding the control region, and is similar in gene content to other hylid mitogenomes. Maximum likelihood analysis, using the mitogenomes of several anurans, indicated *P. tocantins* as closely related to other hylid species.

The paradoxical frog *Pseudis tocantins* Caramaschi and Cruz 1998 is endemic to Brazilian savannas associated with Araguaia and Tocantins river basins (Frost 2018). Interesting features of the paradoxical frogs are related to (1) their extended larval period that results in tadpoles attaining body sizes that are substantially larger than metamorphosed adults (Shaw 1802; Garda et al. 2010; Santana et al. 2016), and (2) the study of sex chromosome differentiation, as *P. tocantins* has highly heteromorphic sex chromosomes (Busin et al. 2008; Gatto et al. 2016). Here, we describe the complete mitochondrial genome of *P. tocantins*.

Total genomic DNA was isolated from the liver of a female *P. tocantins* collected from the type locality of this species (i.e. Porto Nacional, Tocantins state in Brazil – 10°44′38.6″S 48°26′09.2″W), using a standard phenol:chloroform extraction protocol (Sambrook et al. 1989). The remaining tissue is deposited in the tissue collection of the Laboratory of Chromosome Studies of the University of Campinas, Brazil (LabEsC – UNICAMP) under the accession number SMRP 190.15 and the specimen voucher is deposited at the amphibian collection of the Museu de Zoologia Professor Adão José Cardoso at the University of Campinas, Brazil, under the accession number ZUEC 22351. Permission for collecting the specimen was granted by SISBIO (process 45183) and Committee for Ethics in Animal Use of the University of Campinas (CEUA/UNICAMP) (process 3419-1).

A paired-end genomic library was prepared using Nextera DNA Flex Library Prep Kit (Illumina, EUA) and sequenced on an Illumina HiSeq X Ten (Hudson-Alpha Institute for Biotechnology, Alabama, USA). We detected *k*-mers (*k* = 31) occurring at high frequencies among genomic reads using RepARK script (Koch et al. 2014) and default parameters. These high-frequency *k*-mers were then assembled by Velvet (Zerbino and Birney 2008) using default parameters. Genome annotation was performed using MITOS (Bernt et al. 2013). The mitochondrial genome contig was compared with available hylid mitochondrial genomes using BLAST (Altschul et al. 1990). The complete mitochondrial genome of *P. tocantins*, excluding control region, was used in a phylogenetic analysis in comparison with other 13 anurans species. Sequences were aligned using Muscle (Edgar 2004) and used for a Maximum likelihood analysis in RAxML v.0.4.1b (Stamakis 2014), under the GTR + G evolutionary model.

The assembled mitogenome of *P. tocantins* (GenBank accession number MH571152) spanned 15.56 kb, with a GC content of 43.16%. This genome is similar to those of other hylids in size, gene content, and arrangement, with an average nucleotide similarity of 70% when compared to six species from *Hyla* + *Dryophytes*. There are 13 protein-coding genes, 22 tRNA genes, and 2 rRNA genes. These genes are coded in the heavy strand, except for eight tRNA genes (tRNA-Pro, tRNA-Gln, tRNA-Ala, tRNA-Asn, tRNA-Cys, tRNA-Tyr, tRNA-Ser, tRNA-Glu) and one protein-coding gene (ND6).

The resulting phylogenetic tree placed *P. tocantins* as the sister taxon of all extant hylid species analyzed in this paper (Figure 1). This result agrees with the large-scale phylogenetic inferences published thus far, as they place *Pseudis* as a member of the Hylidae family (Faivovich et al. 2005; Wiens et al. 2010; Pyron and Wiens 2011; Duellman et al. 2016).

**Figure 1.**
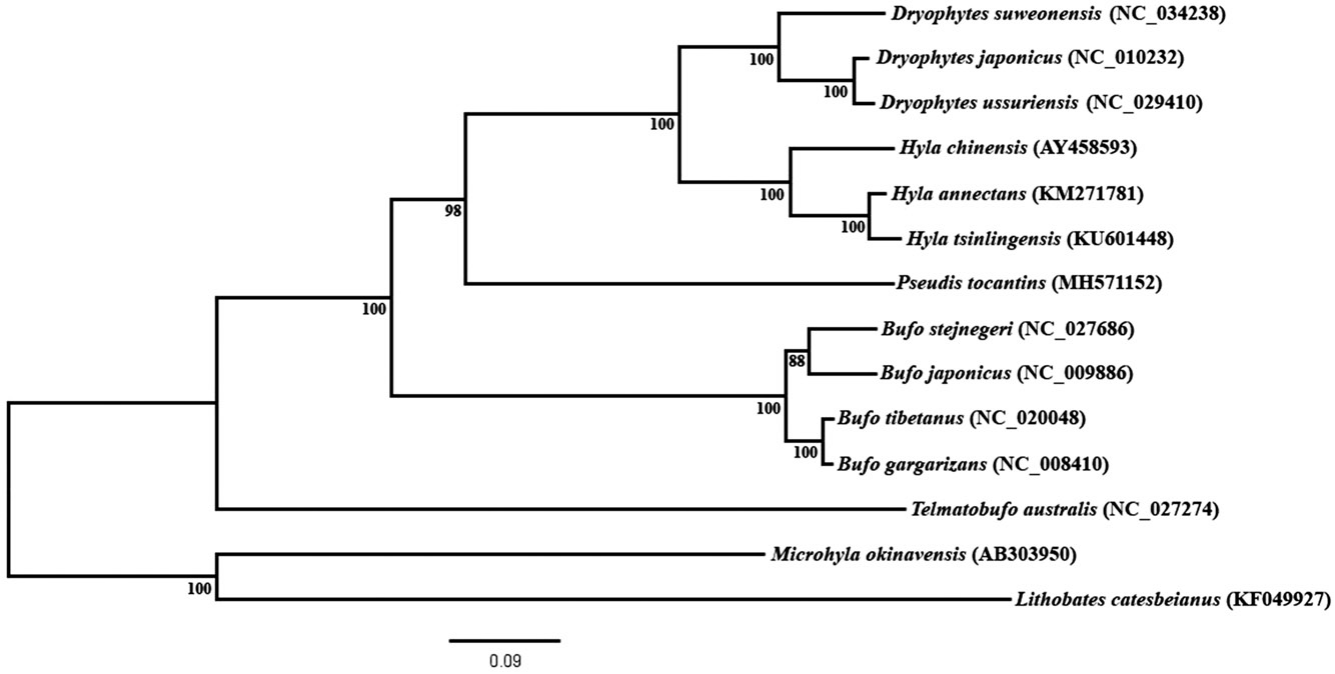
Phylogenetic inference obtained using maximum likelihood based on mitochondrial genomes, excluding control region, of the anuran species *Dryophytes japonicus*, *D. ussuriensis*, (as *Hyla ussuriensis* in Sun et al. 2017), *D. suweonensis* (as *H. suweonensis* in Lee et al. 2017), *Hyla chinensis, H. annectans, Hyla tsinlingensis*, *Pseudis tocantins, Bufo gargarizans, B. tibetanus* (as in Wang et al. 2013), *stejnegeri, B. japonicus, Telmatobufo australis, Microhyla okinavensis* and *Lithobates catesbeianus*. *M. okinavensis* and *L. catesbeianus* were used as outgroups. The phylogenetic inference was constructed under GTR + G evolutionary model. Bootstrap analysis was performed using 1000 pseudoreplicates for node support (numbers at the nodes).
